# Uncontrolled hypertension among patients with comorbidities in sub-Saharan Africa: protocol for a systematic review and meta-analysis

**DOI:** 10.1186/s13643-020-1270-7

**Published:** 2020-01-16

**Authors:** Shukri F. Mohamed, Olalekan A. Uthman, Rishi Caleyachetty, Ivy Chumo, Martin K. Mutua, Gershim Asiki, Paramjit Gill

**Affiliations:** 1 Academic Unit of Primary Care (AUPC) and the NIHR Global Health Research Unit on Improving Health in Slums, University of Warwick, Coventry, UK; 2 Health and Systems for Health Unit, African Population and Health Research Center (APHRC), Nairobi, Kenya; 3 Lown Scholars Program, Department of Global Health and Population, Harvard T.H. Chan School of Public Health, Boston, MA USA; 4 Warwick-Centre for Applied Health Research and Delivery (WCAHRD), Warwick Medical School, University of Warwick, Coventry, UK

**Keywords:** Uncontrolled hypertension, Hypertension, Comorbidities, Sub-Saharan Africa

## Abstract

**Background:**

Uncontrolled hypertension is the most important risk factor and leading cause of cardiovascular diseases. It is predicted that the number of people with hypertension will increase, and a large proportion of this increase will occur in developing countries. The highest prevalence of uncontrolled hypertension is reported in sub-Saharan Africa, and treatment for hypertension is unacceptably low. Hypertension commonly co-exists with comorbidities and this is associated with poorer health outcomes for patients. This review aims to estimate the prevalence of uncontrolled hypertension among patients with comorbidities in sub-Saharan Africa.

**Methods and analysis:**

All published and unpublished studies on the prevalence of uncontrolled hypertension among patients with comorbidities in sub-Saharan Africa will be included. MEDLINE via OVID, Embase, and Web of Science will be searched to identify all relevant articles published from January 2000 to June 2019. Experts in the field will be contacted for unpublished literature, and Open SIGLE will be reviewed for relevant information. No language restriction will be imposed. Two reviewers will select, screen, extract data, and assess the risk of bias while a third reviewer will arbitrate the disagreements. A meta-analysis will be performed on variables that are similar across the included studies. Proportions will be stabilized before estimates are pooled using a random effects model. The presence of publication bias will be assessed using Egger’s test and visual inspection of the funnel plots. This systematic and meta-analysis review protocol will be reported in accordance with the PRISMA-P protocol guidelines. Results will be stratified by country, comorbidity, and geographic region.

**Discussion:**

This systematic review and meta-analysis is expected to quantify the magnitude of uncontrolled hypertension among patients with certain comorbid conditions in sub-Saharan Africa to guide policies and interventions. This review is registered in PROSPERO International Prospective Register of Systematic reviews CRD42019108218.

## Background

Hypertension is one of the leading risk factor causing premature death and disability adjusted life years in the world [1]. More than a billion people in the world had hypertension in 2015 [2] and the highest prevalence (46%) of hypertension was in sub-Saharan Africa (SSA) [3]. It is predicted that the number of people with hypertension will increase to 1.56 billion by 2025, and a large proportion of this increase will occur in developing countries including SSA [4].

Uncontrolled hypertension (UHTN) in SSA is a challenge despite increasing knowledge of hypertension care and the availability of low-cost medications. A 2013 systematic review and meta-analysis showed a very low level of treatment (18%) and a high (97%) pooled prevalence of uncontrolled hypertension in SSA [5]. High uncontrolled rates have economic and public health implications. Morbidities, such as stroke, associated with uncontrolled hypertension are costly to treat and pose a burden to health care systems in SSA that are already weak and strained [6].

At the current rate, achievement of the World Health Organization (WHO) global target of lowering blood pressure by 25% will be challenging to attain by 2025 in SSA [3]. Research has shown that hypertension commonly co-exists with comorbidities such as chronic kidney disease, diabetes, and hypercholesterolemia [7, 8]. Having comorbidities is associated with poorer health outcomes for patients [9], and its management is complex and expensive [10].

There is a dearth of literature on the impact of comorbidities on uncontrolled hypertension in SSA. Previous reviews conducted in SSA have focused on hypertension prevalence, awareness, treatment, and control [5, 11]. The last systematic review that provided an estimate for uncontrolled hypertension in SSA was conducted in 2013 [5]. Since then, many studies have been published and the status of uncontrolled hypertension may have changed; thus, this update is necessary. Therefore, this study aims to conduct a systematic review and meta-analysis to estimate the prevalence of uncontrolled hypertension among patients with comorbidities in sub-Saharan Africa.

## Method and analysis

### Protocol registration

This review is registered in PROSPERO International Prospective Register of Systematic reviews (CRD42019108218) and reported according to Preferred Reporting Items for Systematic reviews and Meta-Analysis protocol (PRISMA-P) guidelines [12]. Additional file 1 shows this in more detail.

### Eligibility criteria

Inclusion criteria:

- Types of studies: All published and unpublished cohort or cross-sectional studies and baseline data from randomized controlled trials conducted in sub-Saharan Africa reporting on the prevalence of high blood pressure/uncontrolled hypertension while on antihypertensive treatment as primary or secondary outcome.
- Participants: Adults’ population with known high blood pressure (hypertension) on any form of antihypertensive medication with any of the following comorbidities: Type 2 diabetes mellitus, dyslipidemia, obesity, chronic kidney disease, stroke, and/or transient ischemic attack, coronary heart disease, heart failure, peripheral vascular disease, atrial fibrillation, depression, or HIV. Study participants should be at least 15 years of age. Essential hypertension (also called primary or idiopathic hypertension) will be defined in accordance with the criteria of the Joint National Committee (JNC) on the Prevention, Detection, Evaluation and Treatment of High Blood Pressure of the US National Heart, Lung and Blood Institute: [1] persistent (seated) systolic blood pressure (SBP) of 140 mmHg or greater or had diastolic blood pressure 90 mmHg or greater regardless of age and sex OR [2] hypertension deducible from the use of antihypertensive drugs or self-reported physician-diagnosed cases. In this study, comorbid conditions that commonly co-exist with hypertension have been identified from Barnett and colleagues’ article on multi-morbidity [13].
- Intervention(s)/exposure(s): On any form of antihypertensive medications.
- Outcome: Prevalence of uncontrolled hypertension among people who report taking antihypertensive treatment and have a comorbid condition/s (Table 1).
- Settings: Hospital and community based studies.
- Publication date: January 1, 2000, to June 2019.
- Language: No language restriction.

**Table 1:** List of 11 conditions included as comorbidity

	Conditions
1	Diabetes
2	Hypercholesterolemia/dyslipidemia/hyperlipidemia/hypertriglyceridemia
3	Obesity
4	Chronic kidney disease
5	Stroke and or transient ischemic attack
6	Coronary heart disease
7	Heart failure
8	Peripheral vascular disease
9	Atrial fibrillation
10	Depression
11	HIV

Exclusion criteria:

- Studies not performed in humans.
- Reviews, commentaries, editorials, letters, and studies without primary data or explicit description of methods, or both.
- Studies only reporting on uncontrolled hypertension but not among patients with the comorbidities of interest.
- Qualitative studies.
- Studies that lack relevant data needed to compute the prevalence of uncontrolled hypertension.
- Studies on pregnancy related hypertension
- Studies in children and adolescent < 15 years.

### Information sources

The following major electronic databases, MEDLINE via OVID, Excerpta Medica Database (Embase), and Web of Science will be searched to identify all relevant articles published from January 1, 2000, to June 15, 2019**.** Further, the reference list of all relevant articles and reviews identified through the search will be scanned to identify additional articles. Unpublished literature will be sought from experts in the field while grey literature such as reports will also be reviewed for relevant information from OpenSIGLE and other organizational websites such as WHO.

### Search strategy

The literature search strategy has been developed using the medical subject headings (MeSH), BOOLEAN operator, and key text words such as “uncontrolled hypertension” OR “hypertension” AND “list of comorbidites,” AND “sub-Saharan Africa.” To ensure maximum sensitivity and precise searches for relevant information, we will add filters such as “Africa South of the Sahara.”

A specific search strategy has been developed with guidance from a librarian with expertise in systematic review searching. The MEDLINE (OVID) search strategy will be adapted to match the syntax and subject headings for the other databases. The search strategy for MEDLINE (OVID), Embase, and Web of Science are displayed in Additional file 2.

### Study records

#### Data management

Based on the inclusion and exclusion criteria, a tool has been developed a priori to guide the screening and selection process. The tool will be piloted and revised before data extraction begins. The search results will be first uploaded to EndNote software first to remove duplicates. The remaining articles will be placed on Rayyan, a mobile and a web-based software program that facilitates the collaboration among the reviewers involved in the screening and selection of studies to be included in the review [14].

#### Selection process

Once data are obtained, two investigators will independently screen the titles and abstracts of articles retrieved from the literature search against the inclusion criteria. Full texts for the eligible titles and/or abstracts including those where there is uncertainty will be obtained for further assessment on whether to include in the study or not. Where necessary, authors will be contacted for additional information to confirm eligibility of studies. Disagreements will be resolved through discussion and when needed there will be arbitration by a third reviewer. Reasons for excluding articles will be recorded.

#### Data collection process

Data will be extracted using a standardized data extraction form. From the studies included, two assessors will independently extract data using the predefined standardized extraction form. Disagreements will be resolved through discussion and when needed there will be arbitration by a third reviewer.

Where there is missing information, the corresponding author of the study will be contacted to request the missing information. A maximum of three emails will be sent to the corresponding author to request for additional information before excluding the study. For studies appearing in more than one published article, we will consider the most recent, comprehensive, and with the largest sample size. For surveys appearing in one article with multiple surveys conducted at different time points, we shall treat each survey as a separate study. For studies that are multi-national, data will be separated to show the estimate at country level.

#### Data items

Data on general information, authors, year, country, and region (Eastern, Western, and Central and Southern Africa), type of publication, study characteristics (study design, setting, sample size, response rate, mean or median age, or age range), data on blood pressure measurements, cut-offs for hypertension used, data on diagnosis of hypertension, information on use of antihypertensive medication/therapy, and prevalence estimates of uncontrolled hypertension among those on treatment will be extracted. Where antihypertensive treatment or prevalence information relevant for estimating uncontrolled hypertension among those on treatment are not available, we will contact the corresponding author of the study to request the missing information. The prevalence of uncontrolled hypertension will be estimated as a percentage of all the participants on treatment with an antihypertensive.

#### Outcomes and prioritization

The primary outcome is the prevalence of uncontrolled hypertension among people who report taking antihypertensive treatment and have a comorbid condition/s in SSA.

#### Risk of bias in individual studies

To assess the risk of bias and quality of studies included in this review, a tool developed by Hoy et al. for prevalence studies will be used [15] (see Additional file 3). The tool contains 11 items; items 1–4 assess the external validity, 5–10 assess the internal validity, and item 11 is a summary of the overall risk by the reviewer based on the responses of the above 10 items which are scored 1 if yes and 0 if no. Studies will be classified as having a low (> 8), moderate [6–8], or high (≤ 5) risk of bias. This exercise will be done by two reviewers and disagreements will be solved by discussion and where necessary by arbitration involving a third reviewer/author.

For each included study, we will estimate the precision (C) or margin of error, considering the sample size (SS) and the observed prevalence (p) of uncontrolled hypertension from the formula:

1 $$ \mathrm{SS}={Z}^2\ast \mathrm{p}\ast \left(1-\mathrm{p}\right)/{C}^2 $$

where *Z* was the *z* value fixed at 1.96 across studies (corresponding to 95% confidence interval). The desirable margin of error is 5% (0.05) or lower.

#### Data synthesis

Crude numerators and denominators from the individual studies will be used to recalculate the study-specific prevalence. Prevalence estimates will be summarized by geographic regions and by comorbidities.

A meta-analysis will be performed on variables that are similar across the included studies. Proportions will be stabilized using the double arcsine transformation [16], and then, a random effects meta-analysis will be performed [17] to determine the pooled estimate of the prevalence of uncontrolled hypertension among patients with comorbid conditions while on treatment across studies in SSA.

Heterogeneity will be explored using Cochrane’s *Q* and quantified by *I*^*2*^ statistics [18]. Subgroup analyses will be performed based on the following: patient characteristics (age categories, sex, education level, socio-economic status), patient comorbidities (diabetes, obesity, chronic kidney disease among others), study design, study setting (hospital vs community), frequently used hypertension cut-offs, regions (Eastern, Western, and Central and Southern Africa), and by Gross National Income (GNI) will be performed to identify the possible sources of heterogeneity. The definitions of the comorbidities of interest will be collected, and those with the same definitions will be analyzed together.

The presence of publication bias will be assessed using Egger’s test and funnel plots [19]. *P* value < 0.10 on the Egger’s test will be considered to be statistically significant for publication bias. Inter-rater agreements between the researchers involved in study selection and those involved in identification of risk of bias will be assessed using *κ* Cohen’s coefficient [20].

All analyses will be performed using “metaprop” routine using Stata version 15 for Windows [21]. Results will be reported as proportions with corresponding 95% confidence intervals (CIs).

## Discussion

This review will be published in accordance with the PRISMA guidelines [22]. The PRISMA flow diagram will be used to record the different phases of the review process [22]. Summary tables will be used to display the data on distribution of uncontrolled hypertension at regional level by variables of interest such as gender, residence, setting, and person level characteristics. Funnel plots will be used to visualize publication bias of the included studies. Forest plots will display the prevalence estimates of uncontrolled hypertension for the included studies as an overall pooled estimate for SSA. Results from this review will inform healthcare providers on the burden of co-existence of UHTN and comorbidities, hence providing evidence that will inform the required changes needed in clinical practice that will support healthcare services in line with patients’ needs. Findings from this review will be shared in conferences, peer review journals, and on social media platforms.

## Supplementary information

**Additional file 1:.** PRISMA-P checklist

**Additional file 2:.** Search strategy

**Additional file 3:.** Assessment of risk of bias template

## Data Availability

Not applicable

## Abbreviations

DBP: Diastolic blood pressure
GNI: Gross National Income
PRISMA-P: Preferred Reporting Items for Systematic reviews and Meta-Analysis protocol
SBP: Systolic blood pressure
SS: Sample size
SSA: Sub-Saharan Africa
UHTN: Uncontrolled hypertension
