# Supplementary material for: Uncontrolled hypertension among patients with comorbidities in sub-Saharan Africa: protocol for a systematic review and meta-analysis
Source: Syst Rev. 2020 Jan 16;9:16. doi: 10.1186/s13643-020-1270-7 (PMC6964205; doi:10.1186/s13643-020-1270-7)
Supplement: Supplementary file 2 — Additional file 2:. Search strategy [file 13643_2020_1270_MOESM2_ESM.docx]

**Additional file 2**

**Medline - search**

1. exp Hypertension/ or hypertension.mp.

2. exp Hypertension/ or uncontrolled hypertension.mp.

3. exp Hypertension/ or uncontrolled blood pressure.mp.

4. high blood pressure.mp. or exp Hypertension/

5. 1 or 2 or 3 or 4

6. type 2 diabetes mellitus.mp. or exp Diabetes Mellitus, Type 2/

7. type 2 diabetes.mp. or exp Diabetes Mellitus, Type 2/

8. exp Diabetes Mellitus, Type 2/ or type II diabetes.mp.

9. dyslipidemia.mp. or exp Dyslipidemias/

10. exp Dyslipidemias/ or dyslipidimia.mp.

11. exp Dyslipidemias/ or dyslipidaemia.mp.

12. Hypercholesterolemia.mp. or exp Hypercholesterolemia/

13. Hypercholesterolaemia.mp. or exp Hypercholesterolemia/

14. Hypercholesterolimia.mp. [mp=title, abstract, original title, name of substance word, subject heading word, floating sub-heading word, keyword heading word, organism supplementary concept word, protocol supplementary concept word, rare disease supplementary concept word, unique identifier, synonyms]

15. hypertriglyceridemia.mp. or exp Hypertriglyceridemia/

16. exp Hypertriglyceridemia/ or hypertriglyceridaemia.mp.

17. hypertriglyceridimia.mp.

18. hyperlipidemia.mp. or exp Hyperlipidemias/

19. exp Hyperlipidemias/ or hyperlipidaemia.mp.

20. hyperlipidimia.mp.

21. obesity.mp. or exp Obesity/

22. chronic kidney disease.mp. or exp Renal Insufficiency, Chronic/

23. stroke.mp. or exp Stroke/

24. transient ischemic attack.mp. or exp Ischemic Attack, Transient/

25. Stroke/ or exp Ischemic Attack, Transient/ or transient ischaemic attack.mp.

26. coronary heart disease.mp. or exp Coronary Disease/

27. Heart failure.mp. or exp Heart Failure/

28. peripheral vascular disease.mp. or exp Peripheral Vascular Diseases/

29. atrial fibrillation.mp. or exp Atrial Fibrillation/

30. depression.mp. or exp Depression/

31. HIV/ or HIV.mp.

32. human immunodeficiency virus.mp. or exp HIV/

33. 6 or 7 or 8 or 9 or 10 or 11 or 12 or 13 or 14 or 15 or 16 or 17 or 18 or 19 or 20 or 21 or 22 or 23 or 24 or 25 or 26 or 27 or 28 or 29 or 30 or 31 or 32

34. (Angola or Benin or Botswana or "Burkina Faso" or "Upper Volta" or Burundi or Urundi or Cameroon or Cameroons or "Cape Verde" or "Central African Republic" or Chad or Comoros or "Comoro Islands" or Comores or Mayotte or Congo or Zaire or "Cote d'Ivoire" or "Ivory Coast" or ("Democratic Republic of the Congo" or Djibouti or "French Somaliland" or Eritrea or Ethiopia or Gabon or "Gabonese Republic" or Gambia or Ghana or "Gold Coast" or Guinea or Kenya or Lesotho or Basutoland or Liberia) or (Madagascar or "Malagasy Republic" or Malawi or Nyasaland or Mali or Mauritania or Mauritius or Mozambique or Namibia or Niger or Nigeria) or (Rwanda or "Sao Tome" or Seychelles or Senegal or "Sierra Leone" or Somalia or "South Africa" or Sudan or Swaziland or Tanzania or Togo or "Togolese Republic" or Uganda or Zambia or Zimbabwe or Rhodesia)).mp. [mp=title, abstract, original title, name of substance word, subject heading word, floating sub-heading word, keyword heading word, organism supplementary concept word, protocol supplementary concept word, rare disease supplementary concept word, unique identifier, synonyms]

35. sub-Saharan africa.mp. or exp "Africa South of the Sahara"/

36. subsaharan africa.mp. or exp "Africa South of the Sahara"/

37. 34 or 35 or 36

38. 5 and 33 and 37

39. limit 38 to (humans and yr="2000 - 2019")

**Embase - search**

1. hypertension.mp. or exp hypertension/

2. exp hypertension/ or uncontrolled hypertension.mp. or exp antihypertensive agent/

3. exp antihypertensive agent/ or exp hypertension/ or uncontrolled blood pressure.mp.

4. high blood pressure.mp. or exp hypertension/

5. 1 or 2 or 3 or 4

6. type 2 diabetes mellitus.mp. or exp non insulin dependent diabetes mellitus/

7. type 2 diabetes.mp. or exp non insulin dependent diabetes mellitus/

8. type II diabetes.mp. or exp non insulin dependent diabetes mellitus/

9. dyslipidemia.mp. or exp dyslipidemia/

10. dyslipidimia.mp.

11. dyslipidaemia.mp. or exp dyslipidemia/

12. exp hypercholesterolemia/ or Hypercholesterolemia.mp.

13. Hypercholesterolaemia.mp. or exp hypercholesterolemia/

14. Hypercholesterolimia.mp.

15. hypertriglyceridemia.mp. or exp hypertriglyceridemia/

16. hypertriglyceridaemia.mp. or exp hypertriglyceridemia/

17. hypertriglyceridimia.mp. or exp hypertriglyceridemia/

18. hyperlipidemia.mp. [mp=title, abstract, original title, name of substance word, subject heading word, floating sub-heading word, keyword heading word, organism supplementary concept word, protocol supplementary concept word, rare disease supplementary concept word, unique identifier, synonyms]

19. hyperlipidaemia.mp. [mp=title, abstract, original title, name of substance word, subject heading word, floating sub-heading word, keyword heading word, organism supplementary concept word, protocol supplementary concept word, rare disease supplementary concept word, unique identifier, synonyms]

20. hyperlipidimia.mp. [mp=title, abstract, original title, name of substance word, subject heading word, floating sub-heading word, keyword heading word, organism supplementary concept word, protocol supplementary concept word, rare disease supplementary concept word, unique identifier, synonyms]

21. obesity.mp. or exp obesity/

22. chronic kidney disease.mp. or exp chronic kidney failure/

23. stroke.mp. or exp cerebrovascular accident/

24. transient ischemic attack.mp. or exp transient ischemic attack/

25. transient ischaemic attack.mp. or exp transient ischemic attack/

26. coronary heart disease.mp. or exp ischemic heart disease/

27. Heart failure.mp. or exp heart failure/

28. peripheral vascular disease.mp. or exp peripheral vascular disease/

29. atrial fibrillation.mp. or exp atrial fibrillation/

30. exp depression/ or depression.mp.

31. HIV.mp. or exp Human immunodeficiency virus/

32. 6 or 7 or 8 or 9 or 10 or 11 or 12 or 13 or 14 or 15 or 16 or 17 or 21 or 22 or 23 or 24 or 25 or 26 or 27 or 28 or 29 or 30 or 31

33. (Angola or Benin or Botswana or "Burkina Faso" or "Upper Volta" or Burundi or Urundi or Cameroon or Cameroons or "Cape Verde" or "Central African Republic" or Chad or Comoros or "Comoro Islands" or Comores or Mayotte or Congo or Zaire or "Cote d'Ivoire" or "Ivory Coast" or ("Democratic Republic of the Congo" or Djibouti or "French Somaliland" or Eritrea or Ethiopia or Gabon or "Gabonese Republic" or Gambia or Ghana or "Gold Coast" or Guinea or Kenya or Lesotho or Basutoland or Liberia) or (Madagascar or "Malagasy Republic" or Malawi or Nyasaland or Mali or Mauritania or Mauritius or Mozambique or Namibia or Niger or Nigeria) or (Rwanda or "Sao Tome" or Seychelles or Senegal or "Sierra Leone" or Somalia or "South Africa" or Sudan or Swaziland or Tanzania or Togo or "Togolese Republic" or Uganda or Zambia or Zimbabwe or Rhodesia)).mp. [mp=title, abstract, original title, name of substance word, subject heading word, floating sub-heading word, keyword heading word, organism supplementary concept word, protocol supplementary concept word, rare disease supplementary concept word, unique identifier, synonyms]

34. sub-Saharan africa.mp. or exp "Africa south of the Sahara"/

35. subsaharan africa.mp. or exp "Africa south of the Sahara"/

36. 33 or 34 or 35

37. 5 and 32 and 36

38. limit 37 to (human and yr="2000 - 2019")

**Web of Science - search**

| # 38 | [**2,114**](http://apps.webofknowledge.com/summary.do;jsessionid=15C3B9506B0B4C7537012C6AC8726B69?product=WOS&doc=1&qid=43&SID=E48A2nzbziMHFAiR8bN&search_mode=CombineSearches&update_back2search_link_param=yes) | #37 AND #33 AND #5  *Indexes=SCI-EXPANDED, SSCI, A&HCI, CPCI-S, CPCI-SSH, ESCI Timespan=2000-2019* | [Edit](http://apps.webofknowledge.com/WOS_AdvancedSearch_input.do?product=WOS&SID=E48A2nzbziMHFAiR8bN&search_mode=AdvancedSearch&replaceSetId=38&editState=init) |  |  |
| --- | --- | --- | --- | --- | --- |
|  | | | | | |
| # 37 | [**421,085**](http://apps.webofknowledge.com/summary.do;jsessionid=15C3B9506B0B4C7537012C6AC8726B69?product=WOS&doc=1&qid=42&SID=E48A2nzbziMHFAiR8bN&search_mode=CombineSearches&update_back2search_link_param=yes) | #36 OR #35 OR #34  *Indexes=SCI-EXPANDED, SSCI, A&HCI, CPCI-S, CPCI-SSH, ESCI Timespan=2000-2019* | [Edit](http://apps.webofknowledge.com/WOS_AdvancedSearch_input.do?product=WOS&SID=E48A2nzbziMHFAiR8bN&search_mode=AdvancedSearch&replaceSetId=37&editState=init) |  |  |
|  | | | | | |
| # 36 | [**301**](http://apps.webofknowledge.com/summary.do;jsessionid=15C3B9506B0B4C7537012C6AC8726B69?product=WOS&doc=1&qid=41&SID=E48A2nzbziMHFAiR8bN&search_mode=AdvancedSearch&update_back2search_link_param=yes) | TS=(subsaharan Africa)  *Indexes=SCI-EXPANDED, SSCI, A&HCI, CPCI-S, CPCI-SSH, ESCI Timespan=2000-2019* | [Edit](http://apps.webofknowledge.com/WOS_AdvancedSearch_input.do?product=WOS&SID=E48A2nzbziMHFAiR8bN&search_mode=AdvancedSearch&replaceSetId=36&editState=init) |  |  |
|  | | | | | |
| # 35 | [**33,673**](http://apps.webofknowledge.com/summary.do;jsessionid=15C3B9506B0B4C7537012C6AC8726B69?product=WOS&doc=1&qid=40&SID=E48A2nzbziMHFAiR8bN&search_mode=AdvancedSearch&update_back2search_link_param=yes) | TS=(sub-Saharan Africa)  *Indexes=SCI-EXPANDED, SSCI, A&HCI, CPCI-S, CPCI-SSH, ESCI Timespan=2000-2019* | [Edit](http://apps.webofknowledge.com/WOS_AdvancedSearch_input.do?product=WOS&SID=E48A2nzbziMHFAiR8bN&search_mode=AdvancedSearch&replaceSetId=35&editState=init) |  |  |
|  | | | | | |
| # 34 | [**407,520**](http://apps.webofknowledge.com/summary.do;jsessionid=15C3B9506B0B4C7537012C6AC8726B69?product=WOS&doc=1&qid=38&SID=E48A2nzbziMHFAiR8bN&search_mode=AdvancedSearch&update_back2search_link_param=yes) | TS=(Angola OR Benin OR Botswana OR "Burkina Faso" OR "Upper Volta" OR Burundi OR Urundi OR Cameroon OR Cameroons OR "Cape Verde" OR "Central African Republic" OR Chad OR Comoros OR "Comoro Islands" OR Comores OR Mayotte OR Congo OR Zaire OR "Cote d'Ivoire" OR "Ivory Coast" OR "Democratic Republic of the Congo" OR Djibouti OR "French Somaliland" OR Eritrea OR Ethiopia OR Gabon OR "Gabonese Republic" OR Gambia OR Ghana OR "Gold Coast" OR Guinea OR Kenya OR Lesotho OR Basutoland OR Liberia OR Madagascar OR "Malagasy Republic" OR Malawi OR Nyasaland OR Mali OR Mauritania OR Mauritius OR Mozambique OR Namibia OR Niger OR Nigeria OR Rwanda OR "Sao Tome" OR Seychelles OR Senegal OR "Sierra Leone" OR Somalia OR "South Africa" OR Sudan OR Swaziland OR Tanzania OR Togo OR "Togolese Republic" OR Uganda OR Zambia OR Zimbabwe OR Rhodesia)  *Indexes=SCI-EXPANDED, SSCI, A&HCI, CPCI-S, CPCI-SSH, ESCI Timespan=2000-2019* | [Edit](http://apps.webofknowledge.com/WOS_AdvancedSearch_input.do?product=WOS&SID=E48A2nzbziMHFAiR8bN&search_mode=AdvancedSearch&replaceSetId=34&editState=init) |  |  |
|  | | | | | |
| # 33 | [**1,764,519**](http://apps.webofknowledge.com/summary.do;jsessionid=15C3B9506B0B4C7537012C6AC8726B69?product=WOS&doc=1&qid=37&SID=E48A2nzbziMHFAiR8bN&search_mode=CombineSearches&update_back2search_link_param=yes) | #32 OR #31 OR #30 OR #29 OR #28 OR #27 OR #26 OR #25 OR #24 OR #23 OR #22 OR #21 OR #20 OR #19 OR #18 OR #17 OR #16 OR #15 OR #14 OR #13 OR #12 OR #11 OR #10 OR #9 OR #8 OR #7 OR #6  *Indexes=SCI-EXPANDED, SSCI, A&HCI, CPCI-S, CPCI-SSH, ESCI Timespan=2000-2019* | [Edit](http://apps.webofknowledge.com/WOS_AdvancedSearch_input.do?product=WOS&SID=E48A2nzbziMHFAiR8bN&search_mode=AdvancedSearch&replaceSetId=33&editState=init) |  |  |
|  | | | | | |
| # 32 | [**85,771**](http://apps.webofknowledge.com/summary.do;jsessionid=15C3B9506B0B4C7537012C6AC8726B69?product=WOS&doc=1&qid=36&SID=E48A2nzbziMHFAiR8bN&search_mode=AdvancedSearch&update_back2search_link_param=yes) | TS=(Human immunodeficiency virus)  *Indexes=SCI-EXPANDED, SSCI, A&HCI, CPCI-S, CPCI-SSH, ESCI Timespan=2000-2019* | [Edit](http://apps.webofknowledge.com/WOS_AdvancedSearch_input.do?product=WOS&SID=E48A2nzbziMHFAiR8bN&search_mode=AdvancedSearch&replaceSetId=32&editState=init) |  |  |
|  | | | | | |
| # 31 | [**272,497**](http://apps.webofknowledge.com/summary.do;jsessionid=15C3B9506B0B4C7537012C6AC8726B69?product=WOS&doc=1&qid=35&SID=E48A2nzbziMHFAiR8bN&search_mode=AdvancedSearch&update_back2search_link_param=yes) | TS=(HIV)  *Indexes=SCI-EXPANDED, SSCI, A&HCI, CPCI-S, CPCI-SSH, ESCI Timespan=2000-2019* | [Edit](http://apps.webofknowledge.com/WOS_AdvancedSearch_input.do?product=WOS&SID=E48A2nzbziMHFAiR8bN&search_mode=AdvancedSearch&replaceSetId=31&editState=init) |  |  |
|  | | | | | |
| # 30 | [**369,094**](http://apps.webofknowledge.com/summary.do;jsessionid=15C3B9506B0B4C7537012C6AC8726B69?product=WOS&doc=1&qid=34&SID=E48A2nzbziMHFAiR8bN&search_mode=AdvancedSearch&update_back2search_link_param=yes) | TS=(depression)  *Indexes=SCI-EXPANDED, SSCI, A&HCI, CPCI-S, CPCI-SSH, ESCI Timespan=2000-2019* | [Edit](http://apps.webofknowledge.com/WOS_AdvancedSearch_input.do?product=WOS&SID=E48A2nzbziMHFAiR8bN&search_mode=AdvancedSearch&replaceSetId=30&editState=init) |  |  |
|  | | | | | |
| # 29 | [**80,988**](http://apps.webofknowledge.com/summary.do;jsessionid=15C3B9506B0B4C7537012C6AC8726B69?product=WOS&doc=1&qid=33&SID=E48A2nzbziMHFAiR8bN&search_mode=AdvancedSearch&update_back2search_link_param=yes) | TS=(atrial fibrillation)  *Indexes=SCI-EXPANDED, SSCI, A&HCI, CPCI-S, CPCI-SSH, ESCI Timespan=2000-2019* | [Edit](http://apps.webofknowledge.com/WOS_AdvancedSearch_input.do?product=WOS&SID=E48A2nzbziMHFAiR8bN&search_mode=AdvancedSearch&replaceSetId=29&editState=init) |  |  |
|  | | | | | |
| # 28 | [**20,886**](http://apps.webofknowledge.com/summary.do;jsessionid=15C3B9506B0B4C7537012C6AC8726B69?product=WOS&doc=1&qid=32&SID=E48A2nzbziMHFAiR8bN&search_mode=AdvancedSearch&update_back2search_link_param=yes) | TS=(peripheral vascular disease)  *Indexes=SCI-EXPANDED, SSCI, A&HCI, CPCI-S, CPCI-SSH, ESCI Timespan=2000-2019* | [Edit](http://apps.webofknowledge.com/WOS_AdvancedSearch_input.do?product=WOS&SID=E48A2nzbziMHFAiR8bN&search_mode=AdvancedSearch&replaceSetId=28&editState=init) |  |  |
|  | | | | | |
| # 27 | [**226,090**](http://apps.webofknowledge.com/summary.do;jsessionid=15C3B9506B0B4C7537012C6AC8726B69?product=WOS&doc=1&qid=31&SID=E48A2nzbziMHFAiR8bN&search_mode=AdvancedSearch&update_back2search_link_param=yes) | TS=(heart failure)  *Indexes=SCI-EXPANDED, SSCI, A&HCI, CPCI-S, CPCI-SSH, ESCI Timespan=2000-2019* | [Edit](http://apps.webofknowledge.com/WOS_AdvancedSearch_input.do?product=WOS&SID=E48A2nzbziMHFAiR8bN&search_mode=AdvancedSearch&replaceSetId=27&editState=init) |  |  |
|  | | | | | |
| # 26 | [**144,037**](http://apps.webofknowledge.com/summary.do;jsessionid=15C3B9506B0B4C7537012C6AC8726B69?product=WOS&doc=1&qid=30&SID=E48A2nzbziMHFAiR8bN&search_mode=AdvancedSearch&update_back2search_link_param=yes) | TS=(coronary heart disease)  *Indexes=SCI-EXPANDED, SSCI, A&HCI, CPCI-S, CPCI-SSH, ESCI Timespan=2000-2019* | [Edit](http://apps.webofknowledge.com/WOS_AdvancedSearch_input.do?product=WOS&SID=E48A2nzbziMHFAiR8bN&search_mode=AdvancedSearch&replaceSetId=26&editState=init) |  |  |
|  | | | | | |
| # 25 | [**1,959**](http://apps.webofknowledge.com/summary.do;jsessionid=15C3B9506B0B4C7537012C6AC8726B69?product=WOS&doc=1&qid=29&SID=E48A2nzbziMHFAiR8bN&search_mode=AdvancedSearch&update_back2search_link_param=yes) | TS=(transient ischaemic attack)  *Indexes=SCI-EXPANDED, SSCI, A&HCI, CPCI-S, CPCI-SSH, ESCI Timespan=2000-2019* | [Edit](http://apps.webofknowledge.com/WOS_AdvancedSearch_input.do?product=WOS&SID=E48A2nzbziMHFAiR8bN&search_mode=AdvancedSearch&replaceSetId=25&editState=init) |  |  |
|  | | | | | |
| # 24 | [**11,095**](http://apps.webofknowledge.com/summary.do;jsessionid=15C3B9506B0B4C7537012C6AC8726B69?product=WOS&doc=1&qid=28&SID=E48A2nzbziMHFAiR8bN&search_mode=AdvancedSearch&update_back2search_link_param=yes) | TS=(transient ischemic attack)  *Indexes=SCI-EXPANDED, SSCI, A&HCI, CPCI-S, CPCI-SSH, ESCI Timespan=2000-2019* | [Edit](http://apps.webofknowledge.com/WOS_AdvancedSearch_input.do?product=WOS&SID=E48A2nzbziMHFAiR8bN&search_mode=AdvancedSearch&replaceSetId=24&editState=init) |  |  |
|  | | | | | |
| # 23 | [**278,508**](http://apps.webofknowledge.com/summary.do;jsessionid=15C3B9506B0B4C7537012C6AC8726B69?product=WOS&doc=1&qid=27&SID=E48A2nzbziMHFAiR8bN&search_mode=AdvancedSearch&update_back2search_link_param=yes) | TS=(stroke)  *Indexes=SCI-EXPANDED, SSCI, A&HCI, CPCI-S, CPCI-SSH, ESCI Timespan=2000-2019* | [Edit](http://apps.webofknowledge.com/WOS_AdvancedSearch_input.do?product=WOS&SID=E48A2nzbziMHFAiR8bN&search_mode=AdvancedSearch&replaceSetId=23&editState=init) |  |  |
|  | | | | | |
| # 22 | [**75,433**](http://apps.webofknowledge.com/summary.do;jsessionid=15C3B9506B0B4C7537012C6AC8726B69?product=WOS&doc=1&qid=26&SID=E48A2nzbziMHFAiR8bN&search_mode=AdvancedSearch&update_back2search_link_param=yes) | TS=(chronic kidney disease)  *Indexes=SCI-EXPANDED, SSCI, A&HCI, CPCI-S, CPCI-SSH, ESCI Timespan=2000-2019* | [Edit](http://apps.webofknowledge.com/WOS_AdvancedSearch_input.do?product=WOS&SID=E48A2nzbziMHFAiR8bN&search_mode=AdvancedSearch&replaceSetId=22&editState=init) |  |  |
|  | | | | | |
| # 21 | [**280,562**](http://apps.webofknowledge.com/summary.do;jsessionid=15C3B9506B0B4C7537012C6AC8726B69?product=WOS&doc=1&qid=25&SID=E48A2nzbziMHFAiR8bN&search_mode=AdvancedSearch&update_back2search_link_param=yes) | TS=(obesity)  *Indexes=SCI-EXPANDED, SSCI, A&HCI, CPCI-S, CPCI-SSH, ESCI Timespan=2000-2019* | [Edit](http://apps.webofknowledge.com/WOS_AdvancedSearch_input.do?product=WOS&SID=E48A2nzbziMHFAiR8bN&search_mode=AdvancedSearch&replaceSetId=21&editState=init) |  |  |
|  | | | | | |
| # 20 | [**5**](http://apps.webofknowledge.com/summary.do;jsessionid=15C3B9506B0B4C7537012C6AC8726B69?product=WOS&doc=1&qid=23&SID=E48A2nzbziMHFAiR8bN&search_mode=AdvancedSearch&update_back2search_link_param=yes) | TS=(hyperlipidimia)  *Indexes=SCI-EXPANDED, SSCI, A&HCI, CPCI-S, CPCI-SSH, ESCI Timespan=2000-2019* | [Edit](http://apps.webofknowledge.com/WOS_AdvancedSearch_input.do?product=WOS&SID=E48A2nzbziMHFAiR8bN&search_mode=AdvancedSearch&replaceSetId=20&editState=init) |  |  |
|  | | | | | |
| # 19 | [**2,534**](http://apps.webofknowledge.com/summary.do;jsessionid=15C3B9506B0B4C7537012C6AC8726B69?product=WOS&doc=1&qid=22&SID=E48A2nzbziMHFAiR8bN&search_mode=AdvancedSearch&update_back2search_link_param=yes) | TS=(hyperlipidaemia)  *Indexes=SCI-EXPANDED, SSCI, A&HCI, CPCI-S, CPCI-SSH, ESCI Timespan=2000-2019* | [Edit](http://apps.webofknowledge.com/WOS_AdvancedSearch_input.do?product=WOS&SID=E48A2nzbziMHFAiR8bN&search_mode=AdvancedSearch&replaceSetId=19&editState=init) |  |  |
|  | | | | | |
| # 18 | [**21,065**](http://apps.webofknowledge.com/summary.do;jsessionid=15C3B9506B0B4C7537012C6AC8726B69?product=WOS&doc=1&qid=21&SID=E48A2nzbziMHFAiR8bN&search_mode=AdvancedSearch&update_back2search_link_param=yes) | TS=(hyperlipidemia)  *Indexes=SCI-EXPANDED, SSCI, A&HCI, CPCI-S, CPCI-SSH, ESCI Timespan=2000-2019* | [Edit](http://apps.webofknowledge.com/WOS_AdvancedSearch_input.do?product=WOS&SID=E48A2nzbziMHFAiR8bN&search_mode=AdvancedSearch&replaceSetId=18&editState=init) |  |  |
|  | | | | | |
| # 17 | [**2**](http://apps.webofknowledge.com/summary.do;jsessionid=15C3B9506B0B4C7537012C6AC8726B69?product=WOS&doc=1&qid=19&SID=E48A2nzbziMHFAiR8bN&search_mode=AdvancedSearch&update_back2search_link_param=yes) | TS=(hypertriglyceridimia)  *Indexes=SCI-EXPANDED, SSCI, A&HCI, CPCI-S, CPCI-SSH, ESCI Timespan=2000-2019* | [Edit](http://apps.webofknowledge.com/WOS_AdvancedSearch_input.do?product=WOS&SID=E48A2nzbziMHFAiR8bN&search_mode=AdvancedSearch&replaceSetId=17&editState=init) |  |  |
|  | | | | | |
| # 16 | [**1,000**](http://apps.webofknowledge.com/summary.do;jsessionid=15C3B9506B0B4C7537012C6AC8726B69?product=WOS&doc=1&qid=18&SID=E48A2nzbziMHFAiR8bN&search_mode=AdvancedSearch&update_back2search_link_param=yes) | TS=(hypertriglyceridaemia)  *Indexes=SCI-EXPANDED, SSCI, A&HCI, CPCI-S, CPCI-SSH, ESCI Timespan=2000-2019* | [Edit](http://apps.webofknowledge.com/WOS_AdvancedSearch_input.do?product=WOS&SID=E48A2nzbziMHFAiR8bN&search_mode=AdvancedSearch&replaceSetId=16&editState=init) |  |  |
|  | | | | | |
| # 15 | [**8,591**](http://apps.webofknowledge.com/summary.do;jsessionid=15C3B9506B0B4C7537012C6AC8726B69?product=WOS&doc=1&qid=17&SID=E48A2nzbziMHFAiR8bN&search_mode=AdvancedSearch&update_back2search_link_param=yes) | TS=(hypertriglyceridemia)  *Indexes=SCI-EXPANDED, SSCI, A&HCI, CPCI-S, CPCI-SSH, ESCI Timespan=2000-2019* | [Edit](http://apps.webofknowledge.com/WOS_AdvancedSearch_input.do?product=WOS&SID=E48A2nzbziMHFAiR8bN&search_mode=AdvancedSearch&replaceSetId=15&editState=init) |  |  |
|  | | | | | |
| # 14 | **0** | TS=(hypercholesterolimia)  *Indexes=SCI-EXPANDED, SSCI, A&HCI, CPCI-S, CPCI-SSH, ESCI Timespan=2000-2019* | [Edit](http://apps.webofknowledge.com/WOS_AdvancedSearch_input.do?product=WOS&SID=E48A2nzbziMHFAiR8bN&search_mode=AdvancedSearch&replaceSetId=14&editState=init) |  |  |
|  | | | | | |
| # 13 | [**3,188**](http://apps.webofknowledge.com/summary.do;jsessionid=15C3B9506B0B4C7537012C6AC8726B69?product=WOS&doc=1&qid=14&SID=E48A2nzbziMHFAiR8bN&search_mode=AdvancedSearch&update_back2search_link_param=yes) | TS=(hypercholesterolaemia)  *Indexes=SCI-EXPANDED, SSCI, A&HCI, CPCI-S, CPCI-SSH, ESCI Timespan=2000-2019* | [Edit](http://apps.webofknowledge.com/WOS_AdvancedSearch_input.do?product=WOS&SID=E48A2nzbziMHFAiR8bN&search_mode=AdvancedSearch&replaceSetId=13&editState=init) |  |  |
|  | | | | | |
| # 12 | [**27,232**](http://apps.webofknowledge.com/summary.do;jsessionid=15C3B9506B0B4C7537012C6AC8726B69?product=WOS&doc=1&qid=13&SID=E48A2nzbziMHFAiR8bN&search_mode=AdvancedSearch&update_back2search_link_param=yes) | TS=(hypercholesterolemia)  *Indexes=SCI-EXPANDED, SSCI, A&HCI, CPCI-S, CPCI-SSH, ESCI Timespan=2000-2019* | [Edit](http://apps.webofknowledge.com/WOS_AdvancedSearch_input.do?product=WOS&SID=E48A2nzbziMHFAiR8bN&search_mode=AdvancedSearch&replaceSetId=12&editState=init) |  |  |
|  | | | | | |
| # 11 | [**4,539**](http://apps.webofknowledge.com/summary.do;jsessionid=15C3B9506B0B4C7537012C6AC8726B69?product=WOS&doc=1&qid=12&SID=E48A2nzbziMHFAiR8bN&search_mode=AdvancedSearch&update_back2search_link_param=yes) | TS=(dyslipidaemia)  *Indexes=SCI-EXPANDED, SSCI, A&HCI, CPCI-S, CPCI-SSH, ESCI Timespan=2000-2019* | [Edit](http://apps.webofknowledge.com/WOS_AdvancedSearch_input.do?product=WOS&SID=E48A2nzbziMHFAiR8bN&search_mode=AdvancedSearch&replaceSetId=11&editState=init) |  |  |
|  | | | | | |
| # 10 | [**6**](http://apps.webofknowledge.com/summary.do;jsessionid=15C3B9506B0B4C7537012C6AC8726B69?product=WOS&doc=1&qid=10&SID=E48A2nzbziMHFAiR8bN&search_mode=AdvancedSearch&update_back2search_link_param=yes) | TS=(dyslipidimia)  *Indexes=SCI-EXPANDED, SSCI, A&HCI, CPCI-S, CPCI-SSH, ESCI Timespan=2000-2019* | [Edit](http://apps.webofknowledge.com/WOS_AdvancedSearch_input.do?product=WOS&SID=E48A2nzbziMHFAiR8bN&search_mode=AdvancedSearch&replaceSetId=10&editState=init) |  |  |
|  | | | | | |
| # 9 | [**25,588**](http://apps.webofknowledge.com/summary.do;jsessionid=15C3B9506B0B4C7537012C6AC8726B69?product=WOS&doc=1&qid=9&SID=E48A2nzbziMHFAiR8bN&search_mode=AdvancedSearch&update_back2search_link_param=yes) | TS=(dyslipidemia)  *Indexes=SCI-EXPANDED, SSCI, A&HCI, CPCI-S, CPCI-SSH, ESCI Timespan=2000-2019* | [Edit](http://apps.webofknowledge.com/WOS_AdvancedSearch_input.do?product=WOS&SID=E48A2nzbziMHFAiR8bN&search_mode=AdvancedSearch&replaceSetId=9&editState=init) |  |  |
|  | | | | | |
| # 8 | [**16,630**](http://apps.webofknowledge.com/summary.do;jsessionid=15C3B9506B0B4C7537012C6AC8726B69?product=WOS&doc=1&qid=8&SID=E48A2nzbziMHFAiR8bN&search_mode=AdvancedSearch&update_back2search_link_param=yes) | TS=(type II diabetes)  *Indexes=SCI-EXPANDED, SSCI, A&HCI, CPCI-S, CPCI-SSH, ESCI Timespan=2000-2019* | [Edit](http://apps.webofknowledge.com/WOS_AdvancedSearch_input.do?product=WOS&SID=E48A2nzbziMHFAiR8bN&search_mode=AdvancedSearch&replaceSetId=8&editState=init) |  |  |
|  | | | | | |
| # 7 | [**173,805**](http://apps.webofknowledge.com/summary.do;jsessionid=15C3B9506B0B4C7537012C6AC8726B69?product=WOS&doc=1&qid=7&SID=E48A2nzbziMHFAiR8bN&search_mode=AdvancedSearch&update_back2search_link_param=yes) | TS=(type 2 diabetes)  *Indexes=SCI-EXPANDED, SSCI, A&HCI, CPCI-S, CPCI-SSH, ESCI Timespan=2000-2019* | [Edit](http://apps.webofknowledge.com/WOS_AdvancedSearch_input.do?product=WOS&SID=E48A2nzbziMHFAiR8bN&search_mode=AdvancedSearch&replaceSetId=7&editState=init) |  |  |
|  | | | | | |
| # 6 | [**94,317**](http://apps.webofknowledge.com/summary.do;jsessionid=15C3B9506B0B4C7537012C6AC8726B69?product=WOS&doc=1&qid=6&SID=E48A2nzbziMHFAiR8bN&search_mode=AdvancedSearch&update_back2search_link_param=yes) | TS=(Type 2 diabetes mellitus)  *Indexes=SCI-EXPANDED, SSCI, A&HCI, CPCI-S, CPCI-SSH, ESCI Timespan=2000-2019* | [Edit](http://apps.webofknowledge.com/WOS_AdvancedSearch_input.do?product=WOS&SID=E48A2nzbziMHFAiR8bN&search_mode=AdvancedSearch&replaceSetId=6&editState=init) |  |  |
|  | | | | | |
| # 5 | [**375,418**](http://apps.webofknowledge.com/summary.do;jsessionid=15C3B9506B0B4C7537012C6AC8726B69?product=WOS&doc=1&qid=5&SID=E48A2nzbziMHFAiR8bN&search_mode=CombineSearches&update_back2search_link_param=yes) | #4 OR #3 OR #2 OR #1  *Indexes=SCI-EXPANDED, SSCI, A&HCI, CPCI-S, CPCI-SSH, ESCI Timespan=2000-2019* | [Edit](http://apps.webofknowledge.com/WOS_AdvancedSearch_input.do?product=WOS&SID=E48A2nzbziMHFAiR8bN&search_mode=AdvancedSearch&replaceSetId=5&editState=init) |  |  |
|  | | | | | |
| # 4 | [**113,713**](http://apps.webofknowledge.com/summary.do;jsessionid=15C3B9506B0B4C7537012C6AC8726B69?product=WOS&doc=1&qid=4&SID=E48A2nzbziMHFAiR8bN&search_mode=AdvancedSearch&update_back2search_link_param=yes) | TS=(high blood pressure)  *Indexes=SCI-EXPANDED, SSCI, A&HCI, CPCI-S, CPCI-SSH, ESCI Timespan=2000-2019* | [Edit](http://apps.webofknowledge.com/WOS_AdvancedSearch_input.do?product=WOS&SID=E48A2nzbziMHFAiR8bN&search_mode=AdvancedSearch&replaceSetId=4&editState=init) |  |  |
|  | | | | | |
| # 3 | [**3,503**](http://apps.webofknowledge.com/summary.do;jsessionid=15C3B9506B0B4C7537012C6AC8726B69?product=WOS&doc=1&qid=3&SID=E48A2nzbziMHFAiR8bN&search_mode=AdvancedSearch&update_back2search_link_param=yes) | TS=(uncontrolled blood pressure)  *Indexes=SCI-EXPANDED, SSCI, A&HCI, CPCI-S, CPCI-SSH, ESCI Timespan=2000-2019* | [Edit](http://apps.webofknowledge.com/WOS_AdvancedSearch_input.do?product=WOS&SID=E48A2nzbziMHFAiR8bN&search_mode=AdvancedSearch&replaceSetId=3&editState=init) |  |  |
|  | | | | | |
| # 2 | [**4,063**](http://apps.webofknowledge.com/summary.do;jsessionid=15C3B9506B0B4C7537012C6AC8726B69?product=WOS&doc=1&qid=2&SID=E48A2nzbziMHFAiR8bN&search_mode=AdvancedSearch&update_back2search_link_param=yes) | TS=(uncontrolled hypertension)  *Indexes=SCI-EXPANDED, SSCI, A&HCI, CPCI-S, CPCI-SSH, ESCI Timespan=2000-2019* | [Edit](http://apps.webofknowledge.com/WOS_AdvancedSearch_input.do?product=WOS&SID=E48A2nzbziMHFAiR8bN&search_mode=AdvancedSearch&replaceSetId=2&editState=init) |  |  |
|  | | | | | |
| # 1 | [**307,652**](http://apps.webofknowledge.com/summary.do;jsessionid=15C3B9506B0B4C7537012C6AC8726B69?product=WOS&doc=1&qid=1&SID=E48A2nzbziMHFAiR8bN&search_mode=GeneralSearch&update_back2search_link_param=yes) | **TOPIC:** (hypertension)  *Indexes=SCI-EXPANDED, SSCI, A&HCI, CPCI-S, CPCI-SSH, ESCI Timespan=2000-2019* |  |  |  |
