# Supplementary material for: Uncontrolled hypertension among patients with comorbidities in sub-Saharan Africa: protocol for a systematic review and meta-analysis
Source: Syst Rev. 2020 Jan 16;9:16. doi: 10.1186/s13643-020-1270-7 (PMC6964205; doi:10.1186/s13643-020-1270-7)
Supplement: Supplementary file 3 — Additional file 3:. Assessment of risk of bias template [file 13643_2020_1270_MOESM3_ESM.docx]

Additional file 3: Assessment of Risk of Bias (RoB)

|  | External validity | Yes/No |  | Internal validity | Yes/No |
| --- | --- | --- | --- | --- | --- |
| 1 | Was the target population representative of the population in relation to relevant variables? |  | 5 | Were data collected directly from the subjects (as opposed to a proxy)? |  |
| 2 | Was the sampling frame a true or close representation of the target population? |  | 6 | Was an acceptable case definition used in the study? |  |
| 3 | Was some form of random selection used to select the sample, OR was a census undertaken? |  | 7 | Was the study instrument that measured the parameter of interest shown to have validity and reliability? |  |
| 4 | Was the likelihood of nonresponse bias minimal in the study? |  | 8 | Was the same mode of data collection used for all subjects? |  |
|  |  |  | 9 | Was the length of the shortest prevalence period for the parameter of interest appropriate? |  |
|  |  |  | 10 | Were the numerator(s) and denominator(s) for the parameter of interest appropriate? |  |
| 11 | Summary item on the overall risk of study bias | | | |  |

*Adapted from Hoy D, Brooks P, Woolf A, Blyth F, March L, Bain C, et al. Assessing risk of bias in prevalence studies: modification of an existing tool and evidence of interrater agreement. Journal of clinical epidemiology. 2012;65(9):934-9.*
